# Memory B-Cell and Antibody Responses Induced by *Plasmodium falciparum* Sporozoite Immunization

**DOI:** 10.1093/infdis/jiu354

**Published:** 2014-06-25

**Authors:** Wiebke Nahrendorf, Anja Scholzen, Else M. Bijker, Anne C. Teirlinck, Guido J. H. Bastiaens, Remko Schats, Cornelus C. Hermsen, Leo G. Visser, Jean Langhorne, Robert W. Sauerwein

**Affiliations:** 1 Department of Medical Microbiology, Radboud university medical center, Nijmegen; 2 Department of Infectious Diseases, Leiden University Medical Center, The Netherlands; 3 Division of Parasitology, MRC National Institute for Medical Research, London, United Kingdom

**Keywords:** antibody, immunization, malaria, memory B-cell, *Plasmodium*, protection, sporozoite

## Abstract

**Background:**

Immunization of healthy volunteers during receipt of chemoprophylaxis with *Plasmodium falciparum* sporozoites (CPS-immunization) induces sterile protection from malaria. Antibody responses have long been known to contribute to naturally acquired immunity against malaria, but their association with sterile protection after whole sporozoite immunization is not well established. We therefore studied the induction and kinetics of malaria parasite antigen-specific antibodies and memory B-cells (MBCs) during CPS-immunization and their correlation with protection from challenge infection.

**Methods:**

We assessed humoral reactivity to 9 antigens representing different stages of the life cycle of *P. falciparum* by performing standardized MBC enzyme-linked immunospot and enzyme-linked immunosorbent assays on peripheral blood mononuclear cells and plasma samples from 38 Dutch volunteers enrolled in 2 randomized controlled clinical trials.

**Results:**

MBCs and antibodies recognizing pre-erythrocytic and cross-stage antigens were gradually acquired during CPS-immunization. The magnitude of these humoral responses did not correlate with protection but directly reflected parasite exposure in CPS-immunization and challenge.

**Conclusions:**

Humoral responses to the malarial antigens circumsporozoite protein, liver-stage antigen-1, apical membrane antigen-1, and merozoite surface protein-1 do not to predict protection from challenge infection but can be used as sensitive marker of recent parasite exposure.

**Clinical Trials Registration:**

NCT01236612 and NCT01218893.

Malaria remains a major global health burden leading to widespread morbidity and mortality, which is mainly caused by *Plasmodium falciparum* [1]. Apicomplexan *Plasmodium* parasites have a complex multistage life cycle, initiated by anopheline mosquitoes depositing sporozoites into the skin of the vertebrate host, which then migrate to the liver, where they establish a clinically silent infection of hepatocytes. After maturation, merozoites egress from hepatocytes into the bloodstream, where they invade and cyclically replicate within erythrocytes. During blood-stage infection, clinical pathology becomes apparent and can be severe. A safe, affordable, and effective vaccine to supplement other intervention strategies would dramatically benefit public health [2], but a vaccine remains elusive despite immense investment of time and money [3], due to our incomplete understanding of protective immunity [4].

Malaria subunit vaccine development has thus far yielded disappointing results, with RTS,S the only vaccine candidate tested in phase 3 clinical trials. This circumsporozoite protein (CSP)–based vaccine showed an encouraging 50% sterile protection in malaria-naive adult volunteers [5] but only reduced clinical and severe disease by 30%–45% in children in malaria-endemic areas [6, 7]. In contrast, use of whole sporozoites as immunogens has the potential to provide humans with sterile protection against malaria in experimental settings. These regimens often use irradiation-attenuated sporozoites (RAS), which cannot complete liver-stage development [8]. However, RAS requires bites by 1000 mosquitoes [9] or at least 5 intravenous injections of 135 000 sporozoites for sterile protection [10]. Chloroquine chemoprophylaxis combined with fully infectious wild-type sporozoites delivered by mosquito bites (hereafter, “CPS-immunization”) provides sterile and long-lasting protection [11, 12] against pre-erythrocytic parasites (sporozoites and liver-stages) [13] and is 20 times more efficient at providing sterile protection than exposure to RAS. One potential reason for this unprecedented efficiency is the fact that, in contrast to irradiation, chloroquine does not affect pre-erythrocytic parasite development [14] but only kills the pathogenic erythrocytic stage. CPS-immunization is therefore an invaluable tool to systematically delineate mechanisms of protective immunity to malaria.

Antibodies play a critical role in preventing infection by a large range of pathogens [15]. Immediately after antigen encounter, antibodies are produced by short-lived plasma cells [16]. Long-term humoral immune memory, however, is only acquired if long-lived antibody-producing plasma cells and memory B-cells (MBCs) are generated [16–18]. MBCs are activated upon antigen re-encounter and rapidly develop into new antibody-producing cells that replenish the plasma cell pool [17]. In malaria, antibodies are mainly known for their ability to control erythrocytic parasites, thereby contributing to clinical immunity [19]. Their possible contribution to sterile, pre-erythrocytic immunity is less established. In the present study, we therefore investigated the generation of malaria-specific MBC and antibody responses in CPS-immunized volunteers, and assessed their association with sterile protection from challenge infection. We found that the magnitude of these responses, predominantly directed against pre-erythrocytic and cross-stage antigens, does not predict sterile protection from challenge infection but is a sensitive indicator of the degree and nature of antigen exposure during immunization.

## MATERIALS AND METHODS

### Human Ethics Statement

Both clinical trials from which samples for this study were obtained received approval by the Central Committee for Research Involving Human Subjects of the Netherlands (approval NL34273.091.10 for study A and approval NL33904.091.10 for study B) and were registered at ClinicalTrials.gov (clinical trials registration: NCT01236612 for study A and NCT01218893 for study B). The study team complied with the Declaration of Helsinki and good clinical practice, including monitoring of data. Volunteers enrolled in both studies provided written informed consent.

### Clinical Trial Design

**Figure 1.**
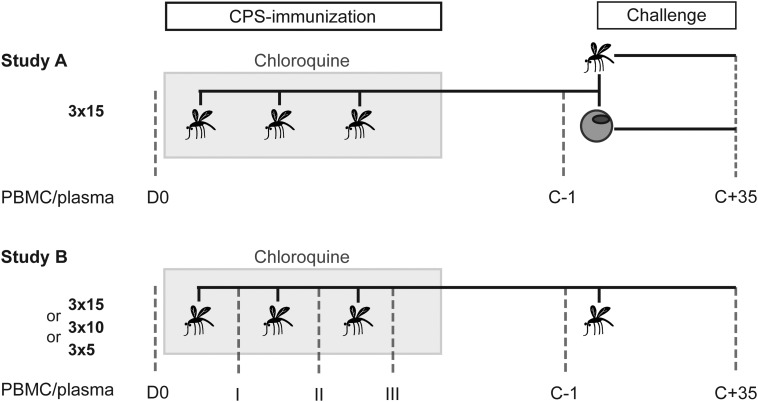
Study design. Samples from 2 clinical chemoprophylaxis and sporozoites (CPS)–immunization trials were analyzed. In study A, volunteers were immunized on 3 occasions, separated by 4-week intervals, with bites of 15 *Plasmodium falciparum*–infected mosquitoes (3 × 15) while receiving a prophylactic regimen of the antimalarial drug chloroquine (gray box). For challenge infection 21 weeks after the last immunization (17 weeks after the final chloroquine dose), immunized volunteers were split into 2 groups, with one receiving *P. falciparum*–parasitized erythrocytes and the other exposed to bites from 5 infective mosquitoes. Plasma and peripheral blood mononuclear cell (PBMC) samples were obtained before immunization (D0), 1 day before challenge (C − 1), and 35 days after challenge (C + 35). In study B, 3 different immunization groups were exposed on 3 occasions, separated by 4-week intervals, to bites from 15 (3 × 15), 10 (3 × 10), or 5 (3 × 5) *P. falciparum*–infected mosquitoes while receiving chloroquine prophylaxis. All groups were challenged 19 weeks after the last immunization (15 weeks after the last chloroquine dose) with 5 *P. falciparum*–infected mosquito bites. Plasma and PBMC samples were collected before immunization (D0); 28 days after the first (I), second (II), and third (III) immunization; 1 day before challenge (C − 1), and 35 days after challenge (C + 35).

To determine the generation of malaria-specific MBC and antibody responses in individuals who received CPS-immunization and those with primary infection, we used peripheral blood mononuclear cells (PBMCs) and plasma samples from 2 single-center randomized controlled clinical trials (Figure 1). In study A [13], volunteers were exposed bites from 15 *P. falciparum*–infected mosquitoes on 3 occasions, which was previously shown to provide sterile protection from future mosquito challenge [11, 12]. Thereafter they were subjected to challenge with either infected mosquito bites or *intravenous* injection of parasitized erythrocytes. Study B [20] was a CPS-immunization dose de-escalation study in which volunteers were immunized by exposure to bites on 3 occasions from either 15, 10, or 5 *P. falciparum*–infected mosquitoes. All immunized and control subjects in this study were subjected to mosquito challenge. This study setup allowed us to longitudinally assess humoral responses in healthy, previously malaria-naive, adult volunteers before and after exposure to different *P. falciparum* life cycle stages in the context of CPS-immunization, in the presence of prophylactic drug levels, and after challenge infection. After challenge, sterile protection was defined as the absence of parasitized erythrocytes on thick blood smears until 21 days after challenge. Volunteers for whom parasites were detected on a thick blood smear were immediately treated with antimalarial drugs, and all remaining volunteers received presumptive drug treatment 21 days after challenge. Importantly, the amount of exposure to blood-stage parasites during both immunization and challenge was characterized for each volunteer retrospectively by quantitative polymerase chain reaction (qPCR) analysis. For further details about study groups, outcomes and sample collection see supplementary information.

### PBMCs and Plasma Samples

PBMCs were isolated by density centrifugation, cryopreserved at 10^7^ cells/mL in fetal calf serum (Gibco)/10% dimethyl sulfoxide (Merck), using Mr Frosty freezing containers (Nalgene), and stored in vapor-phase nitrogen. Plasma samples were stored in aliquots at −20°C and rethawed no more than twice.

### Analysis of MBC and Antibody Responses

The generation of malaria-specific MBCs was assessed by an MBC enzyme-linked immunospot assay [21]. Cryopreserved PBMCs were thawed and stimulated for 5 days with the lectin Pokeweed Mitogen, *Staphylococcus aureus* Protein A, Toll-like receptor 9 ligand ODN 2006, and recombinant human interleukin 10 to promote development of MBCs into antibody-secreting cells (ASCs) [22]. ASCs were then quantified by enzyme-linked immunospot analysis. Levels of malaria antigen–specific antibodies were determined in plasma by a standardized enzyme-linked immunosorbent assay as arbitrary units (AU) in relation to a pool of 100 sera from adults living in an area of Tanzania where malaria is highly endemic [11]. For a detailed description of both methods, see the Supplementary Materials.

### Statistical Analysis

Data were analyzed using GraphPad Prism v6. Differences between values at 2 time points were analyzed by the Wilcoxon matched-pairs signed rank test (2 tailed, nonparametric, and paired). Unmatched data between 2 groups were analyzed by the Mann–Whitney *U* test (2-tailed and nonparametric). Differences between values from >2 times points were analyzed by the Friedman test with the Dunn multiple comparison post hoc test (2 tailed, nonparametric, and paired). Differences between values for ≥2 different groups over time were analyzed by a repeated-measures, mixed-model, 2-way analysis of variance with the Bonferroni post hoc test. Correlations were analyzed by Spearman correlation (nonparametric). Differences with a *P* value of <.05 were considered statistically significant.

## RESULTS

### Humoral Responses Against Pre-erythrocytic and Cross-Stage Malaria Antigens Are Induced by CPS-Immunization

**Figure 2.**
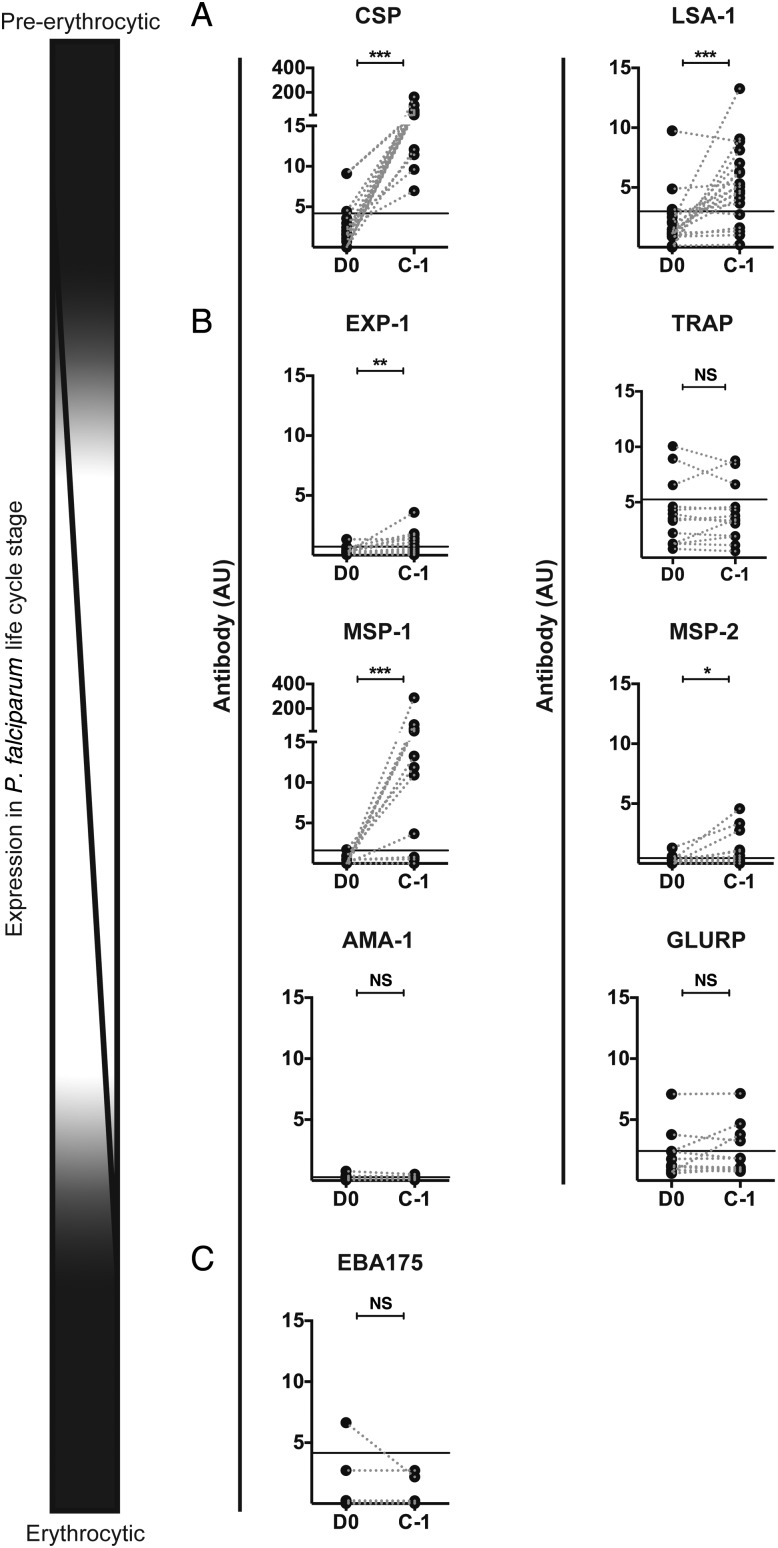
Antibody responses against antigens representing different life cycle stages of the malaria parasite. Antibody responses were determined before immunization (D0) and 19 to 21 weeks after the last chemoprophylaxis and sporozoites (CPS)–immunization (C − 1) for volunteers (14 from study A and 5 from study B) immunized 3 times with bites from 15 infected mosquitoes. Responses are expressed as arbitrary units (AU) for malaria antigens representing different life cycle stages of *Plasmodium falciparum* in the human host (schematically represented by the illustration on the left) (*A*) Circumsporozoite protein (CSP) and liver-stage antigen 1 (LSA-1) are expressed in pre-erythrocytic stages (*B*) Exported protein 1 (EXP-1), thrombospondin-related anonymous protein (TRAP), merozoite surface protein 1 (MSP-1) and MSP-2, apical membrane antigen 1 (AMA-1) and glutamate-rich protein (GLURP) are present in liver and blood-stage parasites (*C*) Erythrocyte binding protein 175 (EBA-175) is exclusively expressed in *P. falciparum*–infected erythrocytes. For study B, responses were assessed for CSP, LSA-1, AMA-1, and MSP-1 only. Data are presented as ladder plots, with each dot representing an individual volunteer and dotted lines connecting values before and after immunization for each volunteer. The black line represents assay background levels (upper 99% confidence interval of the mean of >40 malaria-naive samples tested). Differences between D0 and C − 1 were analyzed using the Wilcoxon matched-pairs signed rank test. *P < .05, **P < .01, and ***P < .001. Abbreviation: NS, not significant.

Malaria-specific antibody and MBC responses induced by the highest-dose regimen of CPS-immunization (bites from 15 infected mosquitoes on 3 occasions; Figure 1) were analyzed for 9 *P. falciparum* antigens representing either the pre-erythrocytic or the erythrocytic part of the *Plasmodium* life cycle. CSP and liver-stage antigen 1 (LSA-1) are exclusively expressed by sporozoites and liver-stage parasites, whereas most proteins, including exported protein 1 (EXP-1), thrombospondin-related anonymous protein (TRAP), merozoite surface protein 1 (MSP-1) and MSP-2, apical membrane antigen 1 (AMA-1), and glutamate-rich protein (GLURP), are most highly abundant in blood-stage parasites but are also expressed in late liver-stages (cross-stage antigens). Erythrocyte binding protein 175 (EBA-175), on the other hand, is only expressed in blood-stage parasites. Between 19 and 21 weeks after the last CPS-immunization (ie, the day before challenge [C − 1]; Figure 1), high antibody titers to the pre-erythrocytic antigens CSP and LSA-1 but no antibodies to the erythrocytic antigen EBA175 were detectable (Figure 2*A* and 2*C*). Antibodies for the cross-stage antigens EXP-1, MSP-1, and MSP-2 were induced, whereas no significant induction of AMA-1, GLURP, and TRAP antibodies was observed (Figure 2*B*). All immunized volunteers developed CSP-specific antibodies (above group background), and antibodies were detectable in 74% of volunteers for LSA-1. Over half (53%) of the volunteers had MSP-1–specific antibodies above background after CPS-immunization, while 42% and 37% developed antibodies against EXP-1 and MSP-2, respectively. Immunization also gave rise to CSP-specific MBCs in 95% of volunteers, and 42% of volunteers also developed MSP-1–specific MBCs, while no MBCs specific for any other selected antigen were observed (data not shown).

### In the Absence of Parasite Exposure, Antimalarial Antibody Responses Contract, but MBCs Are Maintained

**Figure 3.**
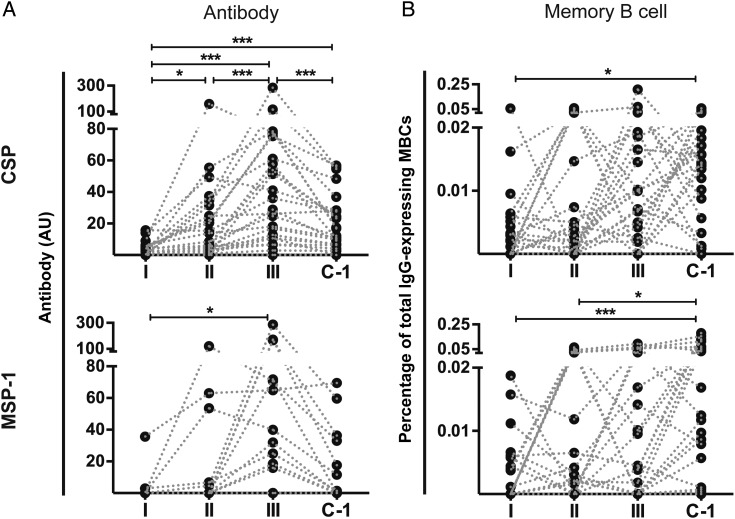
Acquisition kinetics of antimalarial antibody and memory B-cells (MBC) responses. Antibody (*A*; in arbitrary units [AU]) and MBC responses (*B*; presented as the percentage of antigen-specific MBCs of total immunoglobulin G [IgG]–expressing MBCs) for circumsporozoite protein (CSP) and merozoite surface protein 1 (MSP-1) were analyzed 28 days after the first (I), second (II), and third (III) immunization and 19 weeks after the third immunization (C − 1) for all 24 CPS-immunized volunteers in study B. Data were corrected for the volunteers’ background response before immunization and are presented as ladder plots, with each dot representing an individual volunteer and dotted lines connecting the different time points for each volunteer. Differences between the time points were analyzed by the Friedman test with the Dunn multiple comparison post hoc test. *P < .05, **P < .01, and ***P < .001.

**Figure 4.**
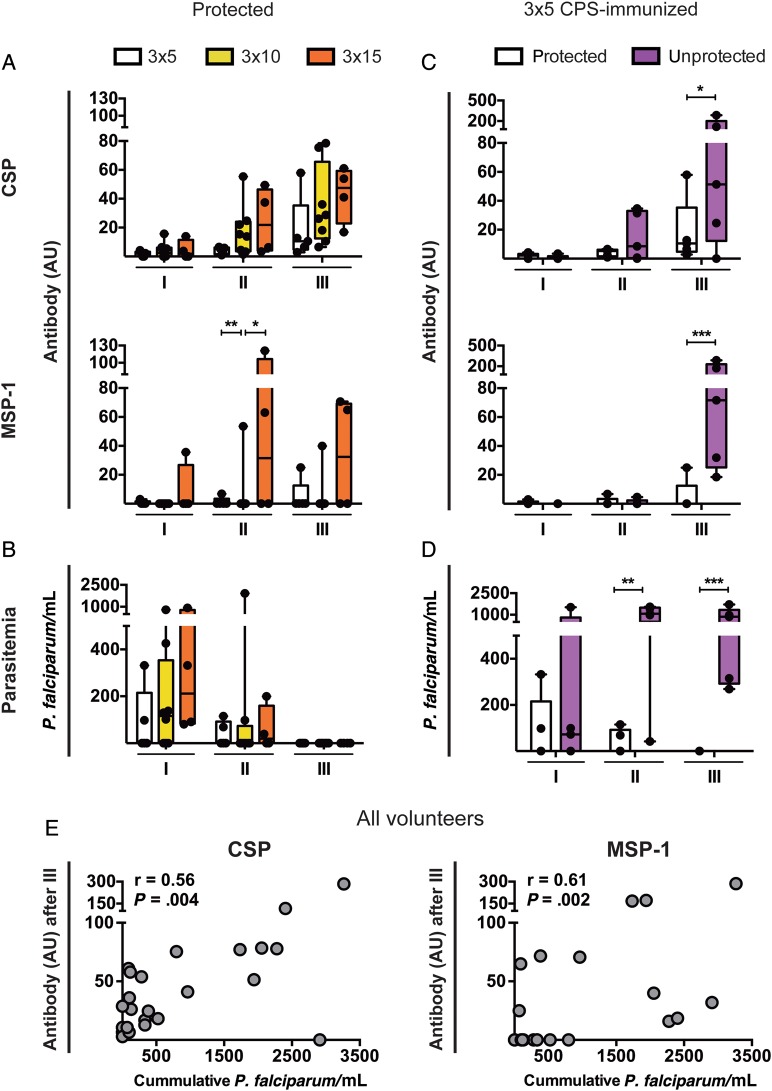
Influence of parasite exposure during immunization on the magnitude of the antibody response and protection status. *A* and *B*, Chemoprophylaxis and sporozoite (CPS)–immunized volunteers from study B protected from mosquito challenge after exposure on 3 occasions, to bites from 15 (3 × 15; orange; n = 4), 10 (3 × 10; yellow; n = 8), or 5 (3 × 5; white; n = 5) *Plasmodium falciparum*–infected mosquitoes. Circumsporozoite protein (CSP)- and merozoite surface protein 1 (MSP-1)–specific antibody levels (in arbitrary units [AU]; *A*) and blood-stage parasitemia (expressed as the number of *P. falciparum–*parasitized erythrocytes per mL of blood, determined by quantitative polymerase chain reaction; *B*) were analyzed 28 days after the first (I), second (II), and third (III) CPS-immunization. *C* and *D*, 3 × 5 CPS-immunization did (white; n = 5) or did not (purple; n = 5) result in protection from subsequent mosquito challenge. *C*, CSP- and MSP-1–specific antibody (in AU) and (*D*) erythrocytic parasitemia (in *P. falciparum*/mL) 28 days after immunizations I, II, and III are displayed. *E*, Correlation between CSP and MSP-1 antibody responses (in AU) 28 days after immunization III and cumulative parasitemia (in *P. falciparum*/mL) over the course of all CPS-immunizations for all study B volunteers. All antibody responses were corrected for the volunteers’ background response before immunization and are presented as individual values (dots) and whisker box plots (box, median with 10th–90th percentile; whiskers, minimum to maximum) and individual values (dots). Differences between groups over time were analyzed by repeated-measures, mixed-model 2-way analysis of variance with the Bonferroni post hoc test. Correlation was analyzed by the Spearman coefficient (r). *P < .05, **P < .01, and ***P < .001.

We next studied the kinetics of acquisition of antibody and MBC responses to the strongest recognized antigens, CSP and MSP-1, over the course of CPS-immunization (Figure 1). CSP- and MSP-1–specific antibody responses increased stepwise after the first, second, and third immunization (Figure 3*A*). Antibody responses peaked after the third immunization but then contracted over the following 15 weeks, until C − 1 (Figure 3*A*). Importantly, a different pattern was observed for the acquisition of MBCs (Figure 3*B*): CSP- and MSP-1-specific MBCs were also acquired in a stepwise fashion during CPS-immunization. However, for the majority of volunteers, they did not contract after the third immunization in the absence of parasite exposure but instead remained stable or even increased during the >4-month period until C − 1. These patterns of CSP- and MSP-1–specific antibody and MBC acquisition were seen irrespective of immunization dose (Figure 4*A* and data not shown).

### The Magnitude of CPS-Induced Humoral Responses Is a Sensitive Reflection of Parasite Exposure but Does Not Predict Sterile Protection

An important question is whether the magnitude of CPS-induced humoral responses to the investigated antigens reflects the degree of parasite exposure and whether they are predictive of sterile protection from challenge infection. We therefore compared immune responses in volunteers who received different immunization doses (Figure 1) and did or did not subsequently experience sterile protection from mosquito challenge.

In volunteers protected from mosquito challenge, there was a trend toward higher antibody responses to CSP with increasing amounts of immunizing infectious mosquito bites, consistent with the different doses of pre-erythrocytic parasites experienced (Figure 4*A*). CPS-immunization with different numbers of infectious mosquito bites also led to a different amount of blood-stage exposure measured by qPCR after the first immunization (Figure 4*B*). This is consistent with the dose-response relation for completed pre-erythrocytic and early erythrocytic development, which is in turn reflected in the induction of MSP-1 antibodies (Figure 4*A*). The level of blood-stage parasitemia was reduced after the second and completely absent after the third CPS-immunization in volunteers subsequently sterilely protected from mosquito challenge (Figure 4*B*).

In the group receiving the lowest immunization dose (bites from 5 mosquitoes on 3 occasions), half of the volunteers were unprotected from mosquito challenge. Unprotected volunteers had significantly higher antibody responses against both CSP and MSP-1 after the third immunization, compared with protected volunteers receiving the same number of immunizing mosquito bites (Figure 4*C*). This was probably a result of the greater parasite exposure during the second and third immunization, reflected by the significantly higher level of blood-stage parasitemia, compared with protected volunteers (Figure 4*D*).

Across all immunized volunteers in this dose de-escalation trial (Figure 1), magnitudes of CSP and MSP-1 antibody responses after the third immunization correlated with the cumulative level of blood-stage parasitemia to which volunteers were exposed over the course of the 3 immunizations (Figure 4*E*).

These data clearly demonstrate that the magnitude of the humoral response against CSP, LSA-1, AMA-1, and MSP-1 after CPS-immunization does not predict protection from challenge infection but accurately reflects parasite exposure (both the number of infectious mosquito bites and the amount of blood-stage parasitemia) during CPS-immunization.

### Boosting of Humoral Responses by Challenge Infection Reflects Exposure to Different *P. falciparum* Life Cycle Stages

**Figure 5.**
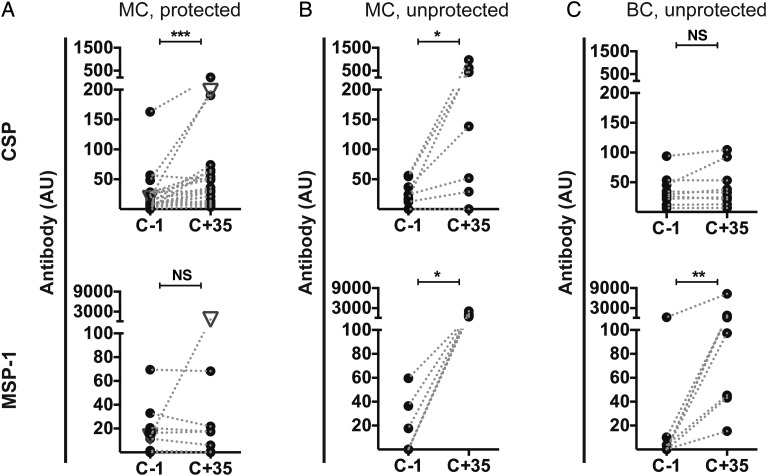
Boosting of antibody levels in chemoprophylaxis and sporozoite (CPS)–immunized volunteers by challenge infection. Antibody levels (in arbitrary units [AU]), corrected for the volunteers’ background before immunization) for circumsporozoite protein (CSP) and merozoite surface protein 1 (MSP-1) in immunized volunteers were determined prior to (C − 1) and 35 days following (C + 35) challenge infection. Each dot represents an individual volunteer for which values before and after challenge are connected by a dotted line. *A,* Mosquito challenge (MC) of 22 protected (experienced pre-erythrocytic parasites only) CPS-immunized volunteers (5 from study A and 17 from study B). The white triangle denotes a volunteer with quantitative polymerase chain reaction–detectable blood-stage parasitemia on day 21 after challenge. *B*, MC of 7 unprotected (experienced both pre-erythrocytic and blood-stage parasites) CPS-immunized volunteers from study B. *C*, Blood-stage challenge (BC; exposure to erythrocytic parasites only) of 9 CPS-immunized volunteers from study A. Differences between time points were analyzed by the Wilcoxon matched-pairs signed rank test. *P < .05, **P < .01, and ***P < .001. Abbreviation: NS, not significant.

Humoral immune responses acquired during CPS-immunization were boosted dependent on parasite exposure by different challenge regimens and protection status (Figure 5 and Supplementary Table 1). A total of 22 immunized volunteers across both clinical trials were protected from mosquito challenge (Figure 1). Since they did not develop blood-stage parasitemia, these volunteers were only exposed to pre-erythrocytic parasites [13]. In line with this, antibody and MBC responses specific for CSP were boosted in these volunteers (Figure 5*A* and Supplementary Table 1), but responses specific for the analyzed cross-and blood-stage antigens were not (Supplementary Table 1). One volunteer, however, who was classified as protected from mosquito challenge on the basis of the absence of parasites on a thick blood smear, was retrospectively found to have developed qPCR-detectable parasitemia (457 parasites/mL) at day 21 after challenge, just prior to receipt of presumptive drug treatment [13]. This small exposure to blood-stage parasites was sufficient to induce a strong boost of MSP-1–specific antibody responses (Figure 5*A*).

Seven CPS-immunized volunteers were unprotected and thus experienced blood-stage parasitemia (as well as pre-erythrocytic parasites) after mosquito challenge (Figure 1). In line with exposure to a broad array of antigens, we found that antibody responses to the pre-erythrocytic antigens CSP and LSA-1 and the cross-stage antigens AMA-1 and MSP-1, as well as MBC responses to CSP, AMA-1, and MSP-1, were boosted in these volunteers (Figure 5*B* and Supplementary Table 1).

Furthermore, 9 CPS-immunized volunteers were subjected to and not protected from injection of parasitized erythrocytes (Figure 1) and, thus, did not experience sporozoites and liver-stage parasites. Antibody responses specific for the cross-stage antigens AMA-1, EXP-1, GLURP, MSP-1, MSP-2, and TRAP were boosted, but no boosting effect was observed for pre-erythrocytic antigens (Figure 5*C* and Supplementary Table 1).

Interestingly, a single mosquito challenge of malaria-naive controls from studies A and B also induced CSP- and MSP-1–reactive antibodies in 80% and 90% of volunteers, respectively (Supplementary Table 2), albeit at lower levels than after mosquito challenge of primed CPS-immunized volunteers. In the majority of volunteers, a single infection was not sufficient to induce a detectable MBC response.

## DISCUSSION

In this study, we provide the first comprehensive and side-by-side analysis of the kinetics and specificity of antibody and MBC responses generated in humans by CPS-immunization and their association with sterile protection. We demonstrate that humoral responses, predominantly against pre-erythrocytic *P. falciparum* antigens, are efficiently generated in a stepwise fashion. The magnitude of these responses does not predict sterile protection from challenge infection but is a sensitive marker of parasite exposure.

We show that CPS-immunization induces strong humoral reactivity to pre-erythrocytic (CSP and LSA-1) and cross-stage (MSP-1) antigens, which is consistent with exposure mainly to pre-erythrocytic parasite antigens during CPS-immunization. Exposure to erythrocytic-stage parasites is limited by chloroquine in this immunization regimen; hence, no humoral responses against AMA-1, GLURP, and EBA175, which are mainly expressed during the erythrocytic stages of the parasite, were detectable. This recognition profile is in line with data from a previous CPS-immunization study, in which antibody reactivity toward CSP but not toward schizont lysate or GLURP was observed in most volunteers [11]. Furthermore, CSP and LSA-1 were also identified as the 2 predominantly recognized antigens by *P. falciparum* protein microarray of specimens from CPS-immunized volunteers [23].

The abundance of *P. falciparum*–specific antibodies and MBCs after CPS-immunization was associated with cumulative parasite exposure, defined as the number of immunizations, the immunization dose, and the level of blood-stage parasitemia experienced. Curiously, correlation of CPS-induced humoral responses with cumulative blood-stage exposure during immunization was not only observed for MSP-1, which is expressed in erythrocytic parasites, but also for CSP, which is often considered a sporozoite-specific antigen. However, it was shown that CSP-expression is not confined to the sporozoite stage but continues until the late stages of liver infection for *P. falciparum* [24], as well as for the rodent parasites *P. berghei* and *P. yoelii* [25, 26]. Since antibody responses are a sensitive reflection of parasite exposure, this indicates that in protected volunteers CPS-induced protective immunity is targeting early pre-erythrocytic stages (sporozoites or early liver-stage parasites), thus reducing the liver-parasite load. Hence, there is shorter/less exposure to the pre-erythrocytic antigen CSP, leading to lower anti-CSP responses in protected volunteers, compared with unprotected volunteers.

Antibody and MBC levels expanded gradually after the first, second, and third CPS-immunization, a kinetic seen independently of the level of parasite exposure during immunization. Antibody titers declined in the absence of parasite exposure between immunization and challenge, likely because of the physiological contraction of short-lived plasma cell responses, whereas MBC levels were largely stably maintained or even increased further. That MBCs are much more long-lived than quickly waning serological responses was recently also reported in African children no longer exposed to malaria [27]. Furthermore, multigravid Ghanaian women showed a much stronger and faster MBC response to pregnancy-restricted *P. falciparum* erythrocyte membrane protein 1 than women in their first pregnancy, also indicating a successful generation and maintenance of humoral immune memory [28]. An intrinsic impairment in the generation of B-cell memory, as suggested previously in individuals with natural exposure to malaria [29, 30], is not apparent in our study.

Challenge infection strongly boosted antibody responses acquired during immunization. This suggests an efficient differentiation of MBCs into ASCs upon antigen re-encounter, even in volunteers experiencing patent blood-stage parasitemia during challenge infection. Therefore, low-grade blood-stage exposure during challenge infection in humans does not lead to an ablation of previously generated MBCs or impaired survival of resulting antibody-producing cells, unlike previous findings for high-level erythrocytic parasitemias in rodent malaria models [31, 32].

Boosting of humoral responses to the different *P. falciparum* antigens was dependent on exposure to different parasite life cycle stages during challenge. Mosquito challenge boosted pre-erythrocytic and cross-stage responses, while exposure to blood-stage parasites boosted responses to cross-stage and erythrocytic antigens. Of note, the subunit vaccine candidate TRAP [33], often classified as a pre-erythrocytic *P. falciparum* protein, was also increased by blood-stage exposure during challenge, strengthening reports of TRAP or TRAP-like protein expression in erythrocytic parasites [34, 35]. We consistently found that MSP-1 antibody responses were strongly boosted in every single immunized volunteer experiencing both full liver-stage and patent erythrocytic parasitemia during challenge infection. That an increase in the MSP-1 antibody level is a reliable indicator even for low-level blood-stage exposure following B-cell priming, in this case by CPS-immunization, was illustrated strikingly in our study: 1 volunteer (classified as protected on the basis of the absence of parasitized erythrocytes on a thick blood smear) was retrospectively shown by qPCR to have had subpatent blood-stage parasitemia [13]. This small exposure to few *P. falciparum*–infected erythrocytes for 1 day only led to a strong boosting of the MSP-1 antibody response. By contrast, MSP-1 antibody levels in volunteers with qPCR-confirmed absence of blood-stage parasitemia after challenge were not boosted. In turn, a lack of boosting of MSP-1–specific antibody responses after challenge infection may be used as an indicator of sterile pre-erythrocytic protection. Taken together, antibody and MBC responses observed in our study depended directly on the amount and life cycle stage of parasite exposure during both CPS-immunization and challenge.

The immunodominant antigens in our study, CSP and MSP1, induced antibodies in the majority of volunteers already after a single CPS-immunization or mosquito challenge and can thus be used as markers of recent parasite exposure. Indeed, CSP antibodies can be used to assess *P. falciparum* transmission dynamics in a naturally exposed population [36]. However, although humoral immune responses during CPS-immunization are efficiently generated, there was no association between the magnitude of humoral responses to CSP, LSA-1, AMA-1, or MSP-1 or any combination of those antigens and protection from challenge infection. CSP-specific antibodies [37, 38] were originally suggested as the mechanism of protection following RAS immunization and have driven the development of the CSP-based malaria vaccine candidate RTS,S. However, despite inducing high CSP-specific antibody responses that can protect human liver-chimeric mice against *P. falciparum* sporozoite challenge [39], RTS,S only confers moderate protection against clinical and severe disease in children and infants in malaria-endemic areas [6, 7]. The association of humoral responses against CSP with sterile protection therefore remains controversial [40–43]. Our results suggest that, after whole sporozoite immunization under chemoprophylaxis, sterile pre-erythrocytic protection may be associated with recognition of novel, less immunodominant antigens. Encouragingly, a pilot protein-microarray study identified 3 novel antigens that were recognized exclusively by protected RAS-immunized volunteers [42]. Analysis of CPS-immunization samples using an extended version of this protein-microarray will help to identify potential new protective antigens. Moreover, protection against malaria may not be associated with humoral responses to one antigen in isolation but instead may be associated with a panel of antigens [44]. In RAS-immunized volunteers, the cumulative response to 19 pre-erythrocytic antigens, rather than reactivity to an individual antigen, differentiated protected from unprotected volunteers [42].

Finally, while the magnitude of individual or combined humoral responses does not predict protection, the actual functional capacity of the resulting antibodies could do so. We have recently shown that antibodies induced by CPS-immunization can reduce the traversal of sporozoites through hepatocytes in vitro, as well as during liver-stage infection and development in vivo in human liver-chimeric mice [45]. However, even antibodies from volunteers with sterile protection could not fully prevent liver-stage infection [45]. In the dose de-escalation CPS-immunization cohort, we have recently shown that sterile protection from challenge infection was associated with induction of higher levels of CD4^+^ T-cells with a cytotoxic phenotype (CD107a^+^) [20]. An integrated analysis of cellular and humoral responses both on antigen recognition and functional level in the same group of volunteers will be crucial to solve the question of a potential immune correlate of sterile protection.

In conclusion, CPS-immunization induced antibody and MBC responses mainly against pre-erythrocytic and cross-stage antigens of *P. falciparum*. Despite their efficient acquisition, the magnitude of acquired humoral immune responses to the set of immunodominant malarial antigens analyzed in this study did not predict sterile protection from challenge infection. Instead, it proved to be a sensitive indicator of the degree and nature of parasite-antigen exposure during both immunization and challenge. The magnitude of acquired antigen-specific humoral responses alone can therefore not be used as a surrogate marker of protective efficacy. Other indicators and assessments of responses to novel antigens are hence necessary to predict protection.

## Supplementary Data

Supplementary materials are available at *The Journal of Infectious Diseases* online (http://jid.oxfordjournals.org). Supplementary materials consist of data provided by the author that are published to benefit the reader. The posted materials are not copyedited. The contents of all supplementary data are the sole responsibility of the authors. Questions or messages regarding errors should be addressed to the author.

Supplementary Data

## References

[1] Lozano R, Naghavi M, Foreman K (2012). Global and regional mortality from 235 causes of death for 20 age groups in 1990 and 2010: a systematic analysis for the Global Burden of Disease Study 2010. Lancet.

[2] Sauerwein RW (2009). Clinical malaria vaccine development. Immunol Lett.

[3] Druilhe P, Barnwell JW (2007). Pre-erythrocytic stage malaria vaccines: time for a change in path. Curr Opin Microbiol.

[4] Riley EM, Stewart VA (2013). Immune mechanisms in malaria: new insights in vaccine development. Nat Med.

[5] Kester KE, Cummings JF, Ofori-Anyinam O (2009). Randomized, double-blind, phase 2a trial of falciparum malaria vaccines RTS,S/AS01B and RTS,S/AS02A in malaria-naive adults: safety, efficacy, and immunologic associates of protection. J Infect Dis.

[6] Bejon P, White MT, Olotu A (2013). Efficacy of RTS,S malaria vaccines: individual-participant pooled analysis of phase 2 data. Lancet Infect Dis.

[7] Olotu A, Fegan G, Wambua J (2013). Four-year efficacy of RTS,S/AS01E and its interaction with malaria exposure. N Engl J Med.

[8] Butler NS, Vaughan AM, Harty JT, Kappe SH (2012). Whole parasite vaccination approaches for prevention of malaria infection. Trends Immunol.

[9] Clyde DF, Most H, McCarthy VC, Vanderberg JP (1973). Immunization of man against sporozite-induced falciparum malaria. Am J Med Sci.

[10] Seder RA, Chang LJ, Enama ME (2013). Protection against malaria by intravenous immunization with a nonreplicating sporozoite vaccine. Science.

[11] Roestenberg M, McCall M, Hopman J (2009). Protection against a malaria challenge by sporozoite inoculation. N Engl J Med.

[12] Roestenberg M, Teirlinck AC, McCall MB (2011). Long-term protection against malaria after experimental sporozoite inoculation: an open-label follow-up study. Lancet.

[13] Bijker EM, Bastiaens GJ, Teirlinck AC (2013). Protection against malaria after immunization by chloroquine prophylaxis and sporozoites is mediated by preerythrocytic immunity. Proc Natl Acad Sci U S A.

[14] Belnoue E, Costa FT, Frankenberg T (2004). Protective T cell immunity against malaria liver stage after vaccination with live sporozoites under chloroquine treatment. J Immunol.

[15] Plotkin SA (2008). Vaccines: correlates of vaccine-induced immunity. Clin Infect Dis.

[16] Tangye SG (2011). Staying alive: regulation of plasma cell survival. Trends Immunol.

[17] Tangye SG, Tarlinton DM (2009). Memory B cells: effectors of long-lived immune responses. Eur J Immunol.

[18] McHeyzer-Williams M, Okitsu S, Wang N, McHeyzer-Williams L (2012). Molecular programming of B cell memory. Nat Rev Immunol.

[19] Cohen S, Mc GI, Carrington S (1961). Gamma-globulin and acquired immunity to human malaria. Nature.

[20] Bijker EM, Teirlinck AC, Schats R (2014). Cytotoxic markers associate with protection against malaria in human volunteers immunized with *Plasmodium falciparum* sporozoites. J Infect Dis.

[21] Crotty S, Aubert RD, Glidewell J, Ahmed R (2004). Tracking human antigen-specific memory B cells: a sensitive and generalized ELISPOT system. J Immunol Methods.

[22] Weiss GE, Ndungu FM, McKittrick N (2012). High efficiency human memory B cell assay and its application to studying *Plasmodium falciparum*-specific memory B cells in natural infections. J Immunol Methods,.

[23] Felgner PL, Roestenberg M, Liang L (2013). Pre-erythrocytic antibody profiles induced by controlled human malaria infections in healthy volunteers under chloroquine prophylaxis. Sci Rep.

[24] Vaughan AM, Mikolajczak SA, Wilson EM (2012). Complete *Plasmodium falciparum* liver-stage development in liver-chimeric mice. J Clin Invest.

[25] Hollingdale MR, Leland P, Leef JL, Leef MF, Beaudoin RL (1983). Serological reactivity of in vitro cultured exoerythrocytic stages of Plasmodium berghei in indirect immunofluorescent or immunoperoxidase antibody tests. Am J Trop Med Hyg.

[26] Singh AP, Buscaglia CA, Wang Q (2007). Plasmodium circumsporozoite protein promotes the development of the liver stages of the parasite. Cell.

[27] Ndungu FM, Olotu A, Mwacharo J (2012). Memory B cells are a more reliable archive for historical antimalarial responses than plasma antibodies in no-longer exposed children. Proc Natl Acad Sci U S A.

[28] Ampomah P, Stevenson L, Ofori MF, Barfod L, Hviid L (2014). Kinetics of B cell responses to *Plasmodium falciparum* erythrocyte membrane protein 1 in ghanaian women naturally exposed to malaria parasites. J Immunol.

[29] Weiss GE, Traore B, Kayentao K (2010). The *Plasmodium falciparum*-specific human memory B cell compartment expands gradually with repeated malaria infections. PLoS Pathog.

[30] Dicko A, Diemert DJ, Sagara I (2007). Impact of a *Plasmodium falciparum* AMA1 vaccine on antibody responses in adult Malians. PLoS One.

[31] Liu XQ, Stacey KJ, Horne-Debets JM (2012). Malaria infection alters the expression of B-cell activating factor resulting in diminished memory antibody responses and survival. Eur J Immunol.

[32] Wykes MN, Zhou YH, Liu XQ, Good MF (2005). Plasmodium yoelii can ablate vaccine-induced long-term protection in mice. J Immunol.

[33] Ewer KJ, O'Hara GA, Duncan CJ (2013). Protective CD8+ T-cell immunity to human malaria induced by chimpanzee adenovirus-MVA immunisation. Nat Commun.

[34] Sharma P, Bharadwaj A, Bhasin VK, Sailaja VN, Chauhan VS (1996). Antibodies to a conserved-motif peptide sequence of the *Plasmodium falciparum* thrombospondin-related anonymous protein and circumsporozoite protein recognize a 78-kilodalton protein in the asexual blood stages of the parasite and inhibit merozoite invasion in vitro. Infect Immun.

[35] Cowan G, Krishna S, Crisanti A, Robson K (1992). Expression of thrombospondin-related anonymous protein in *Plasmodium falciparum* sporozoites. Lancet.

[36] Webster HK, Gingrich JB, Wongsrichanalai C (1992). Circumsporozoite antibody as a serologic marker of *Plasmodium falciparum* transmission. Am J Trop Med Hyg.

[37] Potocnjak P, Yoshida N, Nussenzweig RS, Nussenzweig V (1980). Monovalent fragments (Fab) of monoclonal antibodies to a sporozoite surface antigen (Pb44) protect mice against malarial infection. J Exp Med.

[38] Gysin J, Barnwell J, Schlesinger DH, Nussenzweig V, Nussenzweig RS (1984). Neutralization of the infectivity of sporozoites of Plasmodium knowlesi by antibodies to a synthetic peptide. J Exp Med.

[39] Foquet L, Hermsen CC, van Gemert GJ (2014). Vaccine-induced monoclonal antibodies targeting circumsporozoite protein prevent *Plasmodium falciparum* infection. J Clin Invest.

[40] Gruner AC, Mauduit M, Tewari R (2007). Sterile protection against malaria is independent of immune responses to the circumsporozoite protein. PLoS One.

[41] Kumar KA, Sano G, Boscardin S (2006). The circumsporozoite protein is an immunodominant protective antigen in irradiated sporozoites. Nature.

[42] Trieu A, Kayala MA, Burk C (2011). Sterile protective immunity to malaria is associated with a panel of novel *P. falciparum* antigens. Mole Cell Proteomics.

[43] Doolan DL, Mu Y, Unal B (2008). Profiling humoral immune responses to *P. falciparum* infection with protein microarrays. Proteomics.

[44] John CC, Moormann AM, Pregibon DC (2005). Correlation of high levels of antibodies to multiple pre-erythrocytic *Plasmodium falciparum* antigens and protection from infection. Am J Trop Med Hyg.

[45] Behet MC, Foquet L, van Gemert GJ (2014). Sporozoite immunization of human volunteers under chemoprophylaxis induces functional antibodies against pre-erythrocytic stages of *Plasmodium falciparum*. Malar J.

