# Supplementary material for: Memory B-Cell and Antibody Responses Induced by Plasmodium falciparum Sporozoite Immunization
Source: J Infect Dis. 2014 Jun 25;210(12):1981–90. doi: 10.1093/infdis/jiu354 (PMC4241945; doi:10.1093/infdis/jiu354)
Supplement: Supplementary Data [file supp_210_12_1981__index.html]

Memory B-Cell and Antibody Responses Induced by Plasmodium falciparum Sporozoite Immunization — Supplementary Data 

# Memory B-Cell and Antibody Responses Induced by *Plasmodium falciparum* Sporozoite Immunization

## Supplementary Data

Supplementary Data

**Files in this Data Supplement:**

- Supplementary Data - Pdf file
