# Supplementary material for: Memory B-Cell and Antibody Responses Induced by Plasmodium falciparum Sporozoite Immunization
Source: J Infect Dis. 2014 Jun 25;210(12):1981–90. doi: 10.1093/infdis/jiu354 (PMC4241945; doi:10.1093/infdis/jiu354)
Supplement: Supplementary Data [file supp_jiu354_jiu354supp.pdf]

Supplementary information

Materials and Methods

Clinical trial: study design and sample collection

Study A [1]

Fourteen CPS-immunized volunteers were assigned to receive a challenge with *Pf*-parasitized erythrocytes (blood-challenge BC; n=9) or a sporozoite challenge by infected mosquito bites (MC; n=5). Ten additional volunteers were assigned to two control groups for either BC (n=5) or MC (n=5) (**Fig. 1A**). CPS-immunization was conducted with three times 15 mosquitoes (eight mosquitoes infected with *Pf* strain NF54 and seven mosquitoes infected with *Pf* 3D7 (clone of NF54)). Twenty-one weeks following immunization (17 weeks after discontinuation of chloroquine prophylaxis), all volunteers were subjected to a challenge infection. MC was performed by exposure to bites of five 3D7-infected mosquitoes. BC was conducted by intravenous administration of 1962 viable 3D7 *Pf*-infected erythrocytes. All ten control subjects (MC and BC) and all nine immunized BC volunteers showed parasitized erythrocytes on thick smear and quantitative (q) PCR, whilst the five immunized MC volunteers remained thick smear negative until 21 days after challenge and were then presumptively drug-treated.

Study B [2]

Twenty-nine volunteers were randomly assigned to four groups, three CPS-immunization groups and one control group (**Fig. 1B**). CPS-immunization was carried out using three different numbers of NF54-infected mosquitoes: Study subjects were subjected three times to bites of 15 infected mosquitoes (3x15; n=5), ten infected (and five uninfected) mosquitoes (3x10; n=9) or five infected (and ten uninfected) mosquitoes (3x5; n=10). Control subjects received bites from 15 uninfected mosquitoes (n=5). Therefore, data from 29 challenged subjects were available for analysis. Nineteen weeks following immunization (15 weeks after discontinuation of chloroquine prophylaxis), all volunteers were subjected to a MC challenge infection by bites of five NF54-infected mosquitoes. All five control subjects developed blood-stage parasitemia, as detected by thick smear and qPCR. Of the 24 immunized subjects, sterile protection from MC challenge (defined as blood-stage parasite negative by thick smear until day 21 after challenge) was observed in 4/5 volunteers in the 3x15 group, 8/9 in the 3x10 group and 5/10 of the 3x5 group. The remaining seven CPS-immunized subjects were not protected, but did show a mean delay of 2.5 days in prepatent period of blood-stage parasitemia by both thick smear and qPCR compared to the control subjects. Twenty-one days after challenge, all volunteers remaining thick smear negative were presumptively drug-treated.

Sample collection

Sampling of citrate anti-coagulated peripheral blood for immunological analysis was performed at different time points in Study A and Study B using CPT vacutainers (Becton Dickinson). In Study A, samples were available from D0 (pre-immunization, before onset of chloroquine prophylaxis), day before challenge (C-1)

and 35 days after challenge (C+35). In Study B, additional sampling was performed one month (28 days) after each of the three immunizations (Fig. 1).

Malaria antigens

As representative for pre-erythrocytic antigens we assessed *Pf* CircumSporozoite Protein (CSP) and Liver Stage Antigen 1 (LSA-1) [3]. Erythrocyte Binding Protein 175 (EBA175), purchased from Protein Potential, LLC (9800 Medical Center Drive, Suite A209 Rockville, MD 20852) on the other hand is only expressed in erythrocytic parasites. Most proteins, however, despite being most highly abundant in blood-stage parasites, are also are expressed in late liver-stages (cross-stage antigen). The investigated cross-stage antigens were Apical Membrane Antigen 1 (AMA-1) [4, 5], Exported Protein 1 (EXP-1) [6], Thrombospondin Related Anonymous Protein (TRAP) [7] and the 19 kDa C-terminal region of Merozoite Surface Proteins 1 (MSP-1) [8] and 2 (MSP-2) [9] as well as GLUtamate Rich Protein (GLURP) [10].

Malaria antigen-specific memory B-cell ELISpot assay

Mitogen stimulation

The generation of malaria-specific memory B-cells (MBCs) was assessed by MBC ELISpot assay [11, 12]. Cryopreserved PBMCs were thawed and $1x10^6$cells/ml in RPMI containing 10% FCS, 100U/ml penicillin/streptomycin, 100mM HEPES, 50mM 2-β-Mercaptoethanol and 2mM L-Glutamine (all Invitrogen) were added to $25cm^2$ cell culture flasks (Greiner). To promote development of MBCs into antibody-secreting cells (ASCs), PBMC were stimulated for five days at 37°C and 5% CO_2 with 50ng/ml Pokeweed Mitogen derived from *Phytolacca americana* (Sigma-Aldrich), 1:5000 *Staphylococcus aureus* Protein A, Cowan Strain (Sigma-Aldrich), 2.5µg/ml ODN 2006 (Type B CpG nucleotide-human TLR9 ligand; InvivoGen tlrl-2006 5'-TCGTCGTTTTGTCGTTTTGTCGTT-3') and 25ng/ml recombinant human IL-10 (PeproTech).

ELISpot assay

MultiScreen Filter PVDF Immobilon plates (MSIPS4510, Millipore) pre-treated with 35% Ethanol were coated overnight at 4°C with 10µg/ml monoclonal antibodies to human IgG (clones MT91/145; Mabtech) or 4µg/ml of one of the malaria antigens (all dilutions in PBS). Plates were washed thoroughly and blocked with 1% bovine serum albumin (BSA; Sigma-Aldrich) in RPMI for 2h at 37°C. $4x10^5$ mitogen-stimulated PBMCs per well were seeded in quadruplicates into the malaria antigen-coated filter plates. Anti-IgG coated wells were seeded with 1200 or 4000 cells/well. All time points for one volunteer were measured on the same plate. Filter plates were incubated for 6h at 37°C, 5% CO_2. After washing, immobilized IgG antibody in the proximity of ASCs was detected using polyclonal goat anti-human IgG (Fcγ) alkaline phosphatase (1:1000 in PBST/0.5% FCS; Mabtech) overnight at 4°C. Plates were developed in the dark using alkaline phosphatase substrate BCIP/NBT (Mabtech), rinsed with water, left to dry and stored protected from light until reading using the CTL ImmunoSpot Reader (Cellular Technology Ltd.).

Background correction

Since pre-culture B-cell proportions (percentage of total PBMCs) and post-culture ASCs (per million PBMCs) were highly variable between donors (median with range 5.97 % (1.12-18.32 %) and 31,792 (6625-138,125), respectively), we corrected for inter-individual differences by expressing malaria-specific MBCs as the percentage of total IgG+ MBCs. Group-background levels of MBCs were defined by upper 99% confidence interval of the mean of all time points pre-malaria exposure of all involved volunteers (CSP=0.0054, LSA-1=0.01, TRAP=0.0084, AMA-1=0.01, EXP-1=0.008, MSP1=0.01, MSP-2=0.01, EBA175=0.007, GLURP=0.009). If indicated, MBC responses are presented after individual background correction for each volunteer by subtraction of the average number of spots counted for the specific antigen prior to malaria exposure (immunized individuals D0, control volunteers C-1).

Measurement of malaria-specific plasma antibodies responses

ELISA

Concentrations of malaria antigen-specific antibodies were determined in citrate anti-coagulated plasma in relation to a pool of 100 sera from adults living in a highly endemic area in Tanzania (HIT serum, [13]) by standardized enzyme-linked immunosorbent assay (ELISA). Polystyrene flat-bottom plates (NUNC™ Maxisorp, Thermo Scientific) were coated overnight at 4°C with 1µg/ml malaria antigen in PBS. After washing, plates were blocked with 3% BSA in PBS. Plasma samples were diluted in PBST/1% FCS and analyzed in duplicates. A four-point 1:2 dilution series was carried out for each sample. As a standard, duplicates of pooled HIT serum were included on every plate in a seven-point dilution series. The optimal dilution range was determined prior to the study for every antigen. All time points for one volunteer were measured on the same plate. Two-step detection was performed using biotinylated polyclonal goat anti-human IgG (Fcγ) (Mabtech; 1:1250) and streptavidin-conjugated horseradish peroxidase (HRP, Mabtech, 1:2000). All incubation steps from blocking to detection were carried out for 1h at 37°C in a humidified chamber. Plates were developed at room temperature using HRP substrate (Tetra-methyl-benzidine; tebu-bio); the reaction was stopped using 0.2M H_2SO_4. Spectrophotometrical absorbance at 450nm was measured using the Anthos 2001 ELISA plate reader.

Analysis

The standard curve of HIT serum was plotted on a logarithmic scale and fitted to a power trend line (R^2> 0.99), optical density (OD) measurements for each sample (average of duplicates that were no more than 15% different) were converted to arbitrary units (AU) in relation to HIT serum. For each antigen, undiluted HIT serum was defined to contain 100 AU of IgG directed against this antigen. Dilutions of test samples that did not fall within the linear part of the optical density range of the standard curve were excluded. Group-background levels (in AU) were defined by upper 99% confidence interval of the mean of all time points pre-malaria exposure of all involved volunteers (CSP=4.18, LSA-1=3.0, TRAP=5.25, AMA-1=0.27, EXP-1=0.72, MSP1=1.6, MSP-2=0.45, EBA175=4.16, GLURP=2.42). If indicated, values for malaria antigen-specific antibody responses are presented after individual background correction by subtraction of the average number of AUs for each volunteer for the specific antigen prior to malaria exposure (immunized individuals D0, control volunteers C-1).

Table S1 – Antibody and memory B-cell responses in CPS-immunized volunteers before and after challenge infection

CPS-immunized volunteers (mosquito challenge, protected) [b]

Antibody (AU) [a]

	C-1		C+35		p-value [e]
	median	range	median	range	
CSP	10.72	0-163	30.87	3.8-210	0.0001 *
LSA-1	0.68	0-11.15	0.45	0-10.68	0.03 *
AMA-1	0.00	0-0.09	0.008	0-0.12	0.19
EXP-1	0.01	0-0.99	0.202	0-1.5	0.13
GLURP	0.00	0-0.045	0.00	0-1.05	1.00
MSP-1	0.00	0-69.5	0.00	0-649	0.43
MSP-2	0.00	0-2.05	0.02	0-2.8	0.25
TRAP	0.00	0-0.45	0.00	0-0.73	0.50
EBA175	0.00	0-0.004	0.00	0-0.0	1.00

Memory B-cells (% of total IgG+ MBCs) [a]

	C-1		C+35		p-value [e]
	median	range	median	range	
CSP	0.015	0-0.057	0.035	0-0.29	0.02 *
LSA-1	0.000	0-0.0085	0.000	0-0.0097	0.22
AMA-1	0.004	0-0.020	0.000	0-0.029	0.17
EXP-1	0.000	0-0.0017	0.000	0-0.0072	0.75
GLURP	0.002	0-0.016	0.005	0-0.007	0.63
MSP-1	0.006	0-0.15	0.004	0-0.15	0.73
MSP-2	0.000	0-0.005	0.009	0-0.015	0.25
TRAP	0.002	0-0.012	0.002	0-0.011	0.88
EBA175	0.002	0-0.0040	0.002	0-0.0048	1.00

CPS-immunized volunteers (mosquito challenge, unprotected) [c]

Antibody (AU) [a]

	C-1		C+35		p-value [e]
	median	range	median	range	
CSP	24.40	0-56	138.50	0-965.4	0.03 *
LSA-1	6.06	1.02-16.3	11.18	7.1-99.8	0.02 *
AMA-1	0.02	0-0.14	0.29	0.09-1.43	0.02 *
EXP-1	-	-	-	-	nd
GLURP	-	-	-	-	nd
MSP-1	0.00	0-59.63	979.00	461-2112	0.02 *
MSP-2	-	-	-	-	nd
TRAP	-	-	-	-	nd
EBA175	-	-	-	-	nd

Memory B-cells (% of total IgG+ MBCs) [a]

	C-1		C+35		p-value [e]
	median	range	median	range	
CSP	0.017	0.006-0.04	0.046	0.023-0.11	0.02 *
LSA-1	0.000	0-0.031	0.009	0-0.015	0.44
AMA-1	0.0001	0-0.0076	0.005	0-0.03	0.03 *
EXP-1	-	-	-	-	nd
GLURP	-	-	-	-	nd
MSP-1	0.068	0.012-0.18	0.220	0.07-0.5	0.02 *
MSP-2	-	-	-	-	nd
TRAP	-	-	-	-	nd
EBA175	-	-	-	-	nd

CPS-immunized volunteers (blood-stage challenge, unprotected) [d]

Antibody (AU) [a]

	C-1		C+35		p-value [e]
	median	range	median	range	
CSP	29.22	6.65-94	32.68	6.9-104.6	0.25
LSA-1	5.21	0.44-9.1	4.43	0.72-19.0	0.36
AMA-1	0.00	0-0.078	0.592	0.04-0.87	0.004 *
EXP-1	0.30	0-3.32	3.02	0.27-7.76	0.004 *
GLURP	0.00	0-3.08	0.26	0-5.15	0.008 *
MSP-1	0.00	0-288.4	203.9	15.4-7308	0.004 *
MSP-2	0.66	0-4.32	3.42	0-18.23	0.016 *
TRAP	0.00	0-2.24	0.36	0-12.69	0.019 *
EBA175	0.00	0-0.0067	0.002	0-0.032	0.44

Memory B-cells (% of total IgG+ MBCs) [a]

	C-1		C+35		p-value [e]
	median	range	median	range	
CSP	0.040	0-0.095	0.009	0-0.044	0.08
LSA-1	0.006	0-0.069	0.000	0-0.026	0.22
AMA-1	0.000	0-0.02	0.005	0-0.039	0.15
EXP-1	0.000	0-0.016	0.004	0-0.040	0.38
GLURP	0.003	0-0.016	0.002	0-0.010	0.31
MSP-1	0.005	0-0.019	0.003	0-0.058	0.38
MSP-2	0.000	0-0.022	0.000	0-0.009	0.69
TRAP	0.003	0-0.021	0.000	0-0.0024	0.03 *
EBA175	0.000	0-0.024	0.000	0-0.006	1.00

a. Background corrected for each volunteer by subtraction of individual pre-immunization (D0) values
b. Exposure to pre-erythrocytic stages only; n=5 from Study A and n=17 from Study B (for Study B, responses were assessed for CSP, LSA-1, AMA-1 and MSP-1 only)
c. Exposure to both pre-erythrocytic and blood stages; n=7 from Study B
d. Exposure to blood-stages only; n=9 from Study A
e. Differences between time points were analyzed by Wilcoxon matched-pairs signed rank test. Significant differences are indicated by asterices: * (p<0.05), ** (p<0.01), *** (p<0.001).

Shading: pre-erythrocytic antigens (blue), Cross-stage antigens (green), Blood-stage antigen (red)

Table S2 – Antibody and memory B-cell responses after a single mosquito bite initiated *P. falciparum* infection

Antibody (AU)

Controls (mosquito challenged) [a]

	C-1		C+35		
	median	range	median	range	p-value [b]
CSP	1.13	0-24.72	12.39	0-40.59	0.004 *
LSA-1	1.51	0.73-5.67	2.89	0.54-22.78	0.19
AMA-1	0.16	0.05-0.68	0.140	0.07-0.69	0.77
EXP-1	0.88	0.24-1.18	2.000	0.65-26.67	0.06
GLURP	1.38	0.78-4.19	1.05	0.83-5.46	0.81
MSP-1	0.00	0-0.045	50.42	0-898.1	0.004 *
MSP-2	0.20	0-1.36	0.69	0-7.94	0.50
TRAP	3.32	0.82-7.62	3.29	0.32-6.14	0.44
EBA175	0.06	0.02-0.15	0.09	0.01-0.09	0.44

Memory B-cells (% of total IgG+ MBCs)

	C-1		C+35		
	median	range	median	range	p-value [b]
CSP	0.002	0-0.026	0.002	0-0.036	0.04
LSA-1	0.003	0-0.016	0.005	0-0.016	0.49
AMA-1	0.004	0-0.018	0.002	0-0.012	0.46
EXP-1	0.004	0-0.0094	0.000	0-0.068	0.63
GLURP	0.004	0-0.016	0.000	0-0.009	0.88
MSP-1	0.005	0-0.016	0.005	0-0.029	0.56
MSP-2	0.003	0-0.0094	0.003	0-0.0075	0.88
TRAP	0.005	0-0.008	0.001	0-0.013	0.88
EBA175	0.000	0-0.0078	0.002	0-0.008	0.88

a. Exposure to *Pf* infected mosquito bites (Controls n=5 from Study A and n=5 from Study B, for Study B, responses were assessed for CSP, LSA-1, AMA-1 and MSP-1 only)

b. Differences between time points were analyzed by Wilcoxon matched-pairs signed rank test. Significant differences are indicated by asterices: * ($p<0.05$), ** ($p<0.01$), *** ($p<0.001$).

Shading: pre-erythrocytic antigens (blue), Cross-stage antigens (green), Blood-stage antigen (red)

References

1. Bijker, E.M., G.J. Bastiaens, A.C. Teirlinck, et al., *Protection against malaria after immunization by chloroquine prophylaxis and sporozoites is mediated by preerythrocytic immunity.* Proc Natl Acad Sci U S A, 2013. **110**(19): p. 7862-7.
2. Bijker, E.M., A.C. Teirlinck, R. Schats, et al., *Cytotoxic Markers Associate with Protection against Malaria in Human Volunteers Immunized with Plasmodium falciparum Sporozoites.* J Infect Dis, 2014.
3. Hillier, C.J., L.A. Ware, A. Barbosa, et al., *Process development and analysis of liver-stage antigen 1, a preerythrocyte-stage protein-based vaccine for Plasmodium falciparum.* Infection and immunity, 2005. **73**(4): p. 2109-15.
4. Kocken, C.H., C. Withers-Martinez, M.A. Dubbeld, et al., *High-level expression of the malaria blood-stage vaccine candidate Plasmodium falciparum apical membrane antigen 1 and induction of antibodies that inhibit erythrocyte invasion.* Infection and immunity, 2002. **70**(8): p. 4471-6.
5. Faber, B.W., E.J. Remarque, C.H. Kocken, et al., *Production, quality control, stability and pharmacotoxicity of cGMP-produced Plasmodium falciparum AMA1 FVO strain ectodomain expressed in Pichia pastoris.* Vaccine, 2008. **26**(48): p. 6143-50.
6. Meraldi, V., I. Nebie, R. Moret, et al., *Recognition of synthetic polypeptides corresponding to the N- and C-terminal fragments of Plasmodium falciparum Exp-1 by T-cells and plasma from human donors from African endemic areas.* Parasite immunology, 2002. **24**(3): p. 141-50.
7. Akhouri, R.R., A. Sharma, and P. Malhotra, *Role of Plasmodium falciparum thrombospondin-related anonymous protein in host-cell interactions.* Malaria journal, 2008. **7**: p. 63.
8. Morgan, W.D., M.J. Lock, T.A. Frenkiel, M. Grainger, and A.A. Holder, *Malaria parasite-inhibitory antibody epitopes on Plasmodium falciparum merozoite surface protein-1(19) mapped by TROSY NMR.* Molecular and biochemical parasitology, 2004. **138**(1): p. 29-36.
9. Courtin, D., M. Oesterholt, H. Huismans, et al., *The quantity and quality of African children's IgG responses to merozoite surface antigens reflect protection against Plasmodium falciparum malaria.* PloS one, 2009. **4**(10): p. e7590.
10. Hermsen, C.C., D.F. Verhage, D.S. Telgt, et al., *Glutamate-rich protein (GLURP) induces antibodies that inhibit in vitro growth of Plasmodium falciparum in a phase 1 malaria vaccine trial.* Vaccine, 2007. **25**(15): p. 2930-40.
11. Crotty, S., R.D. Aubert, J. Glidewell, and R. Ahmed, *Tracking human antigen-specific memory B cells: a sensitive and generalized ELISPOT system.* Journal of immunological methods, 2004. **286**(1-2): p. 111-22.
12. Weiss, G.E., F.M. Ndungu, N. McKittrick, et al., *High efficiency human memory B cell assay and its application to studying Plasmodium falciparum-specific memory B cells in natural infections.* Journal of immunological methods, 2012. **375**(1-2): p. 68-74.
13. Roestenberg, M., M. McCall, J. Hopman, et al., *Protection against a malaria challenge by sporozoite inoculation.* The New England journal of medicine, 2009. **361**(5): p. 468-77.
